# Purification, Cloning, Characterization and Essential Amino Acid Residues Analysis of a New ι-Carrageenase from *Cellulophaga* sp. QY3

**DOI:** 10.1371/journal.pone.0064666

**Published:** 2013-05-31

**Authors:** Su Ma, Gaofei Duan, Wengang Chai, Cunliang Geng, Yulong Tan, Lushan Wang, Frédéric Le Sourd, Gurvan Michel, Wengong Yu, Feng Han

**Affiliations:** 1 Key Laboratory of Marine Drugs, Chinese Ministry of Education, Ocean University of China, Qingdao, China; 2 Shandong Provincial Key Laboratory of Glycoscience and Glycotechnology, Ocean University of China, Qingdao, China; 3 Laboratory of Glycobiology, School of Medicine and Pharmacy, Ocean University of China, Qingdao, China; 4 Glycosciences Laboratory, Department of Medicine, Imperial College, London, United Kingdom; 5 The State Key Laboratory of Microbial Technology, Shandong University, Jinan, China; 6 UPMC University Paris 6, Paris, France; 7 CNRS, UMR 7139 Marine Plants and Biomolecules, Station Biologique de Roscoff, Brittany, France; National Institute for Medical Research, Medical Research Council, United Kingdom

## Abstract

ι-Carrageenases belong to family 82 of glycoside hydrolases that degrade sulfated galactans in the red algae known as ι-carrageenans. The catalytic mechanism and some substrate-binding residues of family GH82 have been studied but the substrate recognition and binding mechanism of this family have not been fully elucidated. We report here the purification, cloning and characterization of a new ι-carrageenase CgiA_Ce from the marine bacterium *Cellulophaga* sp. QY3. CgiA_Ce was the most thermostable carrageenase described so far. It was most active at 50°C and pH 7.0 and retained more than 70% of the original activity after incubation at 50°C for 1 h at pH 7.0 or at pH 5.0–10.6 for 24 h. CgiA_Ce was an endo-type ι-carrageenase; it cleaved ι-carrageenan yielding neo-ι-carrabiose and neo-ι-carratetraose as the main end products, and neo-ι-carrahexaose was the minimum substrate. Sequence analysis and structure modeling showed that CgiA_Ce is indeed a new member of family GH82. Moreover, sequence analysis of ι-carrageenases revealed that the amino acid residues at subsites −1 and +1 were more conserved than those at other subsites. Site-directed mutagenesis followed by kinetic analysis identified three strictly conserved residues at subsites −1 and +1 of ι-carrageenases, G228, Y229 and R254 in CgiA_Ce, which played important roles for substrate binding. Furthermore, our results suggested that Y229 and R254 in CgiA_Ce interacted specifically with the sulfate groups of the sugar moieties located at subsites −1 and +1, shedding light on the mechanism of ι-carrageenan recognition in the family GH82.

## Introduction

Carrageenans are sulfated linear polysaccharides of D-galactose extracted from certain red seaweeds, with repeating disaccharide sequences of alternating 3-linked β-D-galactopyranose (β-Gal, G-unit) and 4-linked α-D-galactopyranose (α-Gal, D-unit) [1]. Further classification of carrageenans is based on the occurrence of a 3,6-anhydrobridge in the α-Gal residues (A-unit) and on the degree and pattern of sulfation in the disaccharide repeating unit, e.g. κ-, ι- and λ-carrageenan contain the disaccharide repeating units A-G4S, A2S-G4S and D2S,6S-G2S, respectively [2]. Due to the excellent physical/chemical properties, carrageenans are widely utilized as gelling and stabilizing agents in the food industry and as releasing modifiers of drugs or as pelletizing agents in pharmaceutical industry [3], [4].

Carrageenans are also important carbon sources for heterotrophic marine bacteria, which degrade cell walls of marine red algae by secreting specific glycoside hydrolases (GHs), referred to as carrageenases [5]. Based on their substrate specificity, these enzymes are named as κ-, ι- and λ-carrageenases, respectively. All described carrageenases are endo-hydrolases and cleave β-1,4 glycosidic bonds of carrageenans yielding oligosaccharide products of the neocarrabiose type [6]–[8]. Based on amino acid sequences, these enzymes belong to distinct GH families, κ-carrageenases form a subfamily within the family GH16 [6], ι-carrageenases define the unrelated family GH82 [7], while λ-carrageenases constitute a new GH family [8], although it has not been yet classified in the carbohydrate-active enzymes (CAZy) database [9]. Up to now, nine gene sequences of family GH82 ι-carrageenases have been found, but only four of corresponding proteins have been purified and characterized [7], [10]–[12]. The optimal temperatures for these four ι-carrageenase activities were between 40 and 50°C [7], [10]. However, the thermal and pH stabilities of family GH82 ι-carrageenases have not been studied. In light of the crystal structure of the ι-carrageenase from *Alteromonas fortis* (CgiA_Af), which was the only one published for family GH82, some catalytic and substrate-binding site residues have been proposed [11]. Furthermore, five key catalytic residues have been identified by site-directed mutagenesis and subsequent kinetic analysis for CgiA_Af [12]. However, the substrate recognition and binding mechanism of this GH family have not been experimentally studied.

Our preliminary experiments showed that the supernatant of marine bacterium *Cellulophaga* sp. QY3 had degradation activity towards both κ- and ι-carrageenans (unpublished data). In the present work, we have purified and characterized a new ι-carrageenase and cloned its encoding gene from *Cellulophaga* sp. QY3. Moreover, using 3D structure modeling, consensus sequence analysis, site-directed mutagenesis and kinetic analysis, we have investigated the essential roles of three residues (G228, Y229 and R254) in CgiA_Ce, which are strictly conserved in family GH82.

## Results

### Purification of the Extracellular ι-Carrageenase from *Cellulophaga* sp. QY3

**Figure 1:**
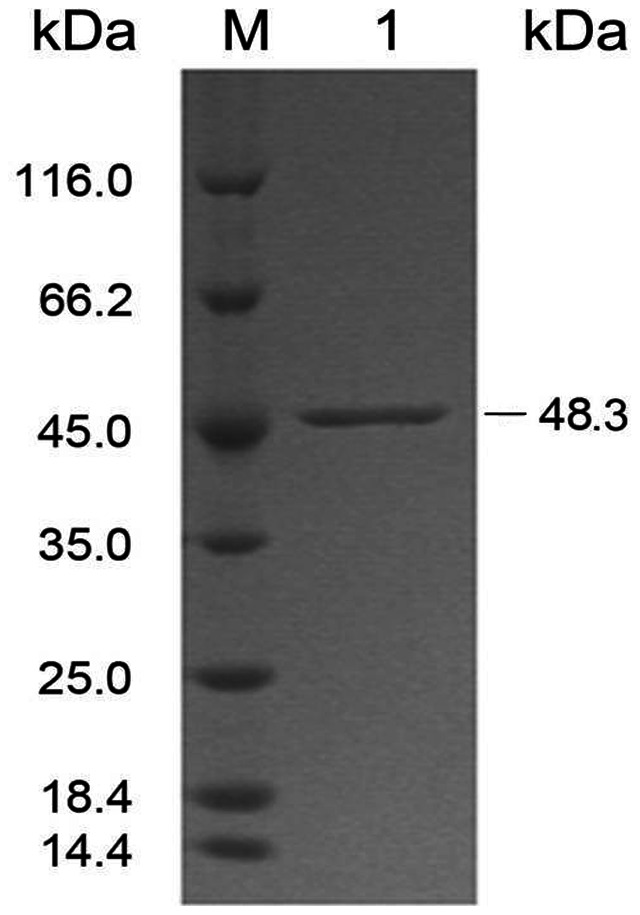
SDS-PAGE of ι-carrageenase CgiA_Ce purified from *Cellulophaga* sp. QY3. Lane M, protein molecular weight marker (Fermentas): lysozyme (14.4 kDa),β-lactoglobulin (18.4 kDa),rease bsp981 (25.0 kDa),lactate dehydrogenase (35.0 kDa),ovalbumin (45.0 kDa),bovine serum albumin (66.2 kDa),β-galactosidase (116.0 kDa); lane 1, purified ι-carrageenase CgiA_Ce.

The ι-carrageenase CgiA_Ce obtained from the culture supernatant of *Cellulophaga* sp. QY3 was purified at electrophoretic homogeneity by a three-step protocol: ammonium sulfate precipitation, hydrophobic interaction and size exclusion chromatography (SEC), typically resulting in a 31-fold purification with a 7.4% recovery (Table 1). The purified enzyme gave a single band with an apparent molecular mass of 48 kDa in SDS-PAGE under reducing condition (Figure 1), consistent with the result estimated by SEC under non-reducing condition (data not shown). Consequently, the purified enzyme is a monomeric protein under the condition used for SEC.

**Table 1:** Purification of ι-carrageenase CgiA_Ce from *Cellulophaga* sp. QY3.

Purification step	Total activity (U)	Total protein (mg)	Specific activity (U/mg)	Purification (fold)	Yield (%)
Culture supernatant	23667	2000	11.8	1	100
(NH_4_)_2_SO_4_ supernatant	22182	1613	13.8	1.2	94
Phenyl Sepharose HP	4263	15	284.2	24	18
Superdex 75	1756	4.8	365.9	31	7.4

### Biochemical Characterization of CgiA_Ce

**Figure 2:**
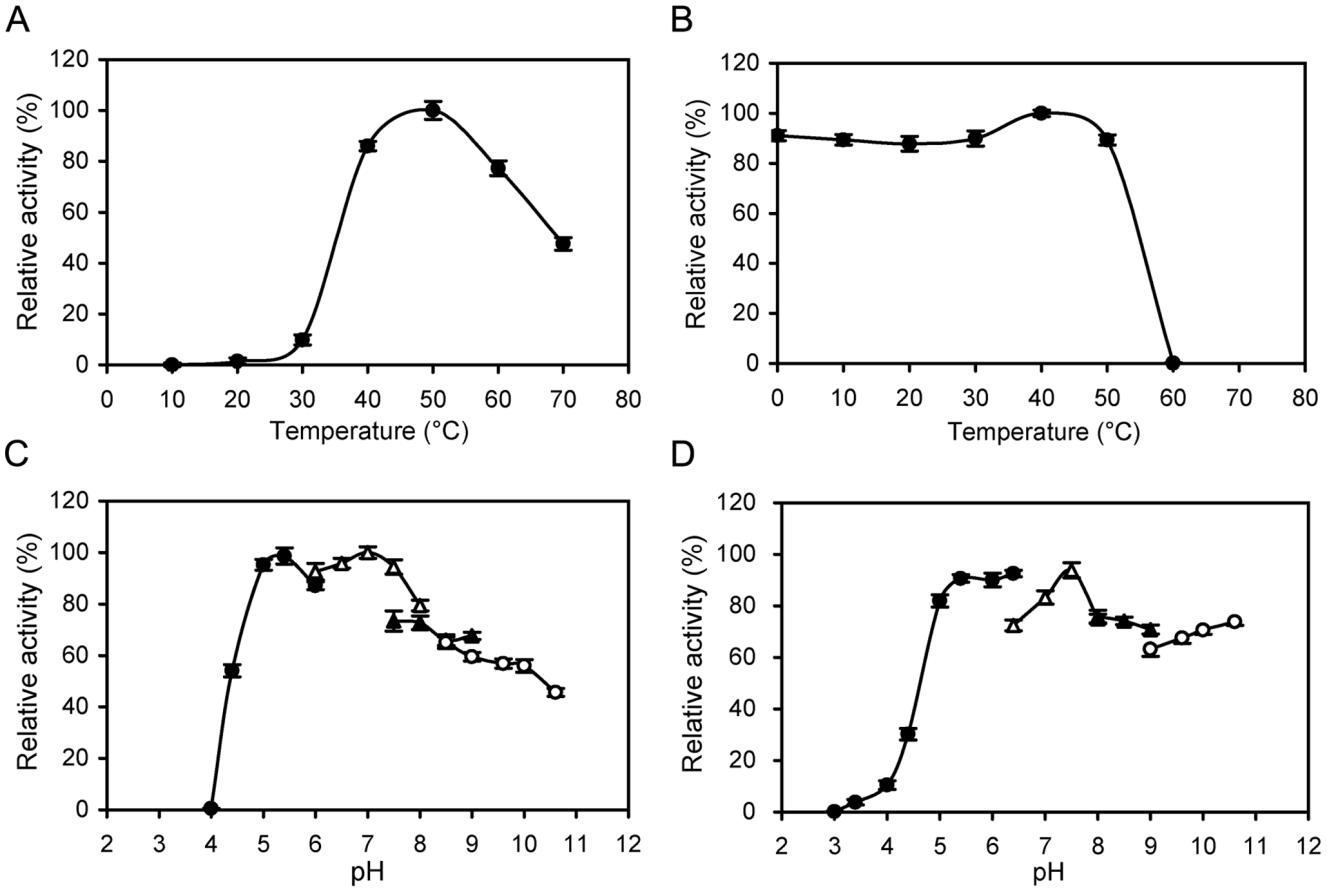
Effects of temperature and pH on CgiA_Ce activity. The values are shown as percentages of the enzyme activity of a fresh sample of ι-carrageenase measured at pH 7.0 and 50°C. (A) The effect of temperature on enzyme activity of CgiA_Ce. (B) Thermostability of the purified CgiA_Ce. (C) The effect of pH on enzyme activity of CgiA_Ce. The buffers used were 50 mM citrate acid buffer (pH 3.0–6.0; closed circles), phosphate buffer (pH 6.0–8.0; open triangles), Tris-HCl buffer (pH 7.5–9.0; closed triangles) and glycine-NaOH buffer (pH 8.6–10.6; open circles). (D) pH stability of the purified CgiA_Ce. Residual activities after incubation at various pHs for 24 h at 4°C were assayed at pH 7.0 and 50°C.

CgiA_Ce was most active at 50°C (Figure 2A) and pH 7.0 (Figure 2C). It retained more than 80% of the original activity after incubation at 50°C for 1 h (Figure 2B). Greater than 70% of the original activity was maintained after treating the enzyme at pH 5.0–10.6 and 4°C for 24 h (Figure 2D). Compared to all known carrageenases, CgiA_Ce showed the highest thermostability and the widest pH stability.

The purified enzyme showed a strict substrate specificity since it was active only on ι-carrageenan but not on either κ- or λ-carrageenan, or on any other polysaccharides such as agar, alginate, cellulose and starch. Cations Na^+^ and K^+^ increased the activity of CgiA_Ce significantly. Highest activity was observed in the presence of 500 mM NaCl or 10 mM KCl (Table 2). Most di- and trivalent metal cations tested reduced the enzyme activity slightly (Table 2). SDS (1 mM) had no effect on the activity of CgiA_Ce.

**Table 2:** Effects of reagents on activity of CgiA_Ce.

Reagent added	Concentration (mM)	Relative activity (%)	Reagent added	Concentration (mM)	Relative activity (%)
none	–	100.0±1.9	(NH_4_)_2_SO_4_	1	85.9±0.7
NaCl	10	111.4±2.2		10	78.6±1.4
	100	126.1±1.8		100	38.3±0.5
	300	134.5±2.5	LiCl	1	93.7±1.3
	500	147.2±3.1	NH_4_Cl	1	97.7±2.0
	700	143.9±1.6	CuCl_2_	1	60.5±0.9
KCl	1	120.4±1.7	BaCl_2_	1	81.9±1.2
	5	129.9±1.1	MnCl_2_	1	52.3±0.8
	10	140.6±0.9	ZnCl_2_	1	87.9±1.1
	50	136.1±2.1	NiCl_2_	1	82.8±0.9
	100	112.8±0.8	CaCl_2_	1	93.9±2.1
	300	109.4±1.1	MgCl_2_	1	85.5±1.7
EDTA	1	92.6±0.9	AlCl_3_	1	80.7±1.1
SDS	1	98.8±1.0	FeCl_3_	1	87.3±0.8

### Analysis of Hydrolysis Pattern and Products of CgiA_Ce

**Figure 3:**
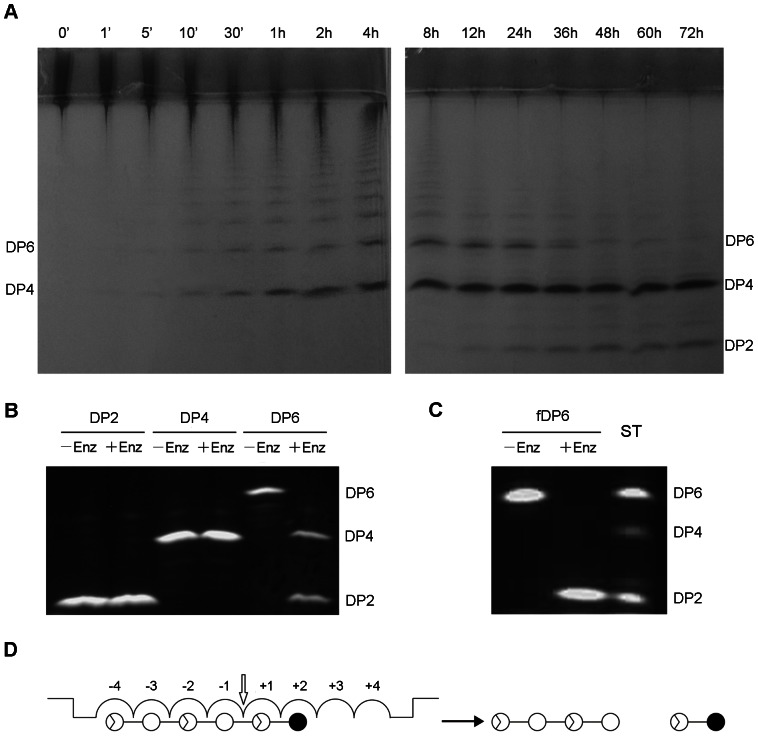
Hydrolysis patterns of CgiA_Ce. DP2: neo-ι-carrabiose; DP4: neo-ι-carratetraose; DP6: neo-ι-carrahexaose; fDP6: fluorescence labeled neo-ι-carrahexaose. (A) FACE analysis of hydrolysate of ι-carrageenan. To visualize the polysaccharides and their hydrolysis products, gels were stained in a 0.2% Alcian Blue solution. (B) FACE analysis of hydrolysis products of neo-carraoligosaccharides (-Enz: enzyme free; +Enz: CgiA_Ce added). (C) PAGE analysis of hydrolysis products of labeled neo-ι-carrahexaose (-Enz: enzyme free; +Enz: CgiA_Ce added). The lane marked ST was loaded with a mixture of DP2, DP4 and DP6 as the standards. (D) Schematic representation of the hydrolysis pattern determined for CgiA_Ce. White circles represent β-linked D-galactose units; white circles with triangular inserts represent α-linked 3,6-anhydro-D-galactose units; black circles represent the reducing ends. The β-1,4 bond cleaved during catalytic hydrolysis is marked with an arrow.

**Figure 4:**
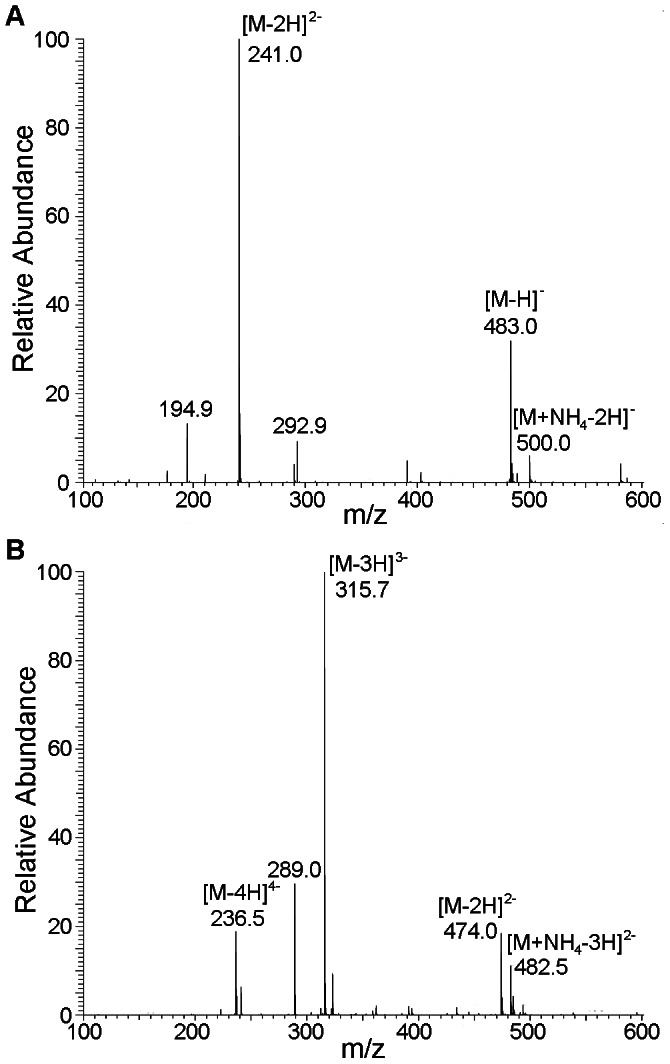
ESI–MS mass spectra of the main hydrolysis products of ι-carrageenan by CgiA_Ce. (A) Bio-Gel P6 fraction F1; (B) Bio-Gel P6 fraction F2.

In the initial hydrolysis stage, there was a rapid reduction of ι-carrageenan polysaccharide and an increase of oligosaccharides with various degrees of polymerization (DPs), indicating that CgiA_Ce behaved as an endo-type hydrolase (Figure 3A). The enzyme tended to preferentially release small ι-carrageenan fragments, suggesting a processive behavior consistent with other reported carrageenases [11], [13]. For a detailed analysis of the hydrolytic characteristics of CgiA_Ce, the main products of the CgiA_Ce were isolated by SEC on a Bio-Gel P6 column (Figure S1). Two main fractions (F1 and F2) were analyzed by negative-ion electrospray mass spectrometry (ESI-MS) (Figure 4) and NMR spectroscopy (Figure S2). The mass spectrum of F1 (Figure 4A) exhibited major ions at m/z 241.0, 483.0 and 500.0, corresponding to the molecular ions [M-2H]^2−^, [M-H]^-^and [M+NH_4_–2H]^-^, respectively, of a ι-carrageenan disaccharide with a composition of A2S_1_.G4S_1_ (484 Da). The mass spectrum of F2 (Figure 4B) showed major ions at m/z 236.5, 315.7, 474.0 and 482.5 as [M-4H]^4−^, [M-3H]^3−^, [M-2H]^2−^, and [M+NH_4_–3H]^2−^, respectively, indicating a tetrasaccharide with a composition of A2S_2_.G4S_2_ (950 Da). The sequence of the tetrasaccharide F2 was obtained by ^1^H- and ^13^C-NMR analyses (Figure S2). In the anomeric proton region of ^1^H-NMR spectrum (Figure S2A), the signal at 5.33 ppm was from H1(α) of the reducing terminal 3-linked G4S and that at 5.28 ppm was from H1(α) of the non-reducing A2S residue, in a complete agreement with the previous report on NMR study of neo-ι-carra-oligosaccharides [14]. The carbon signals corroborated with the assignment made by ^1^H-NMR, e.g. C1 of the reducing terminal G4S at 91.58(α) and 95.90(β) ppm, and C1α of the non-reducing A2S at 91.23 ppm. The sequence of the disaccharide F1 was similarly assigned (spectra not shown).

These results unambiguously demonstrated that the degradation products were of the neo-ι-carra-types. Neo-ι-carra-biose and tetraose, A2S-G4S and A2S-G4S-A2S-G4S, rather than the ι-carra-type (G4S-A2S) were obtained, indicating that CgiA_Ce cleaved β-1,4 linkages of ι-carrageenan.

### The Substrate Binding Subsites of CgiA_Ce

**Figure 5:**
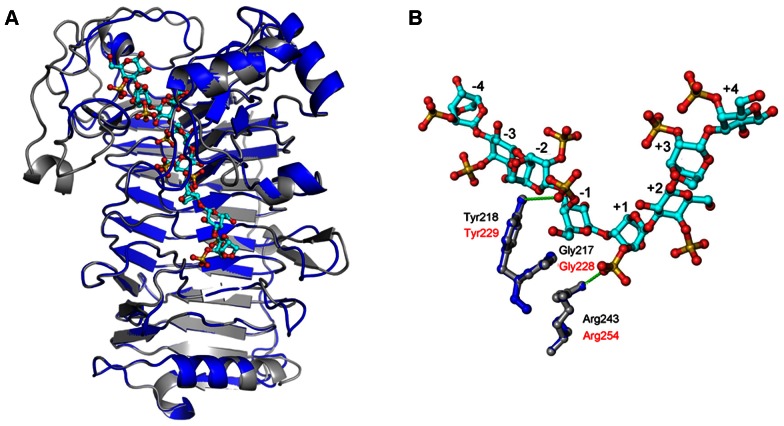
Three-dimensional model of CgiA_Ce. (A) Overall structures of CgiA_Af (gray) and CgiA_Ce (blue). The ι-carrageenan oligosaccharides are shown as balls and sticks. (B) Three conserved amino acid residues of CgiA_Af (gray) and CgiA_Ce (blue) complexed with carrageenan oligosaccharides at subsites −4 to +4.

To determine the number of substrate binding subsites in the active tunnel of CgiA_Ce, we compared the degrading capability of CgiA_Ce on oligosaccharide substrates with defined sizes (DP2 to DP6, Figure 3B). Purified neo-ι-carrabiose and neo-ι-carratetraose were not further degraded by CgiA_Ce even under more forcing conditions (high enzyme concentration and prolonged incubation time). Neo-ι-carrahexaose was the shortest chain that can be recognized and cleaved by CgiA_Ce, producing di- and tetrasaccharide (Figure 3B). The specific activity for hexasaccharide was only 70% of that for polysaccharide, indicating that the number of subsites for CgiA_Ce should be more than six. Based on 3D structural modeling, the active site architecture of CgiA_Ce was considered to be the same as that of CgiA_Af (Figure 5A). Taken together, we deduced that the number of subsites for CgiA_Ce was eight.

To determine the direction and location of the substrate in the active site of CgiA_Ce, the reducing end of the minimum substrate hexasaccharide was tagged with a fluorescent label 7-amino-1,3-naphthalenedisulfonic acid (AGA) by reductive-amination. The AGA tag linked to the hexasaccharides does not substantially alter the mode of binding of substrates to the enzyme [15]. Degradation of fluorescence labeled hexasaccharide gave rise to the production of labeled disaccharide but not labeled tetrasaccharide (Figure 3C), indicating that the hexasaccharide bound to subsites −4 to +2 leading to the cleavage of the glycosidic bond between the second and the third galactose residues from the reducing end (Figure 3D).

### Cloning and Sequence Analysis of *cgiA_Ce* Gene

Translation of the possible open reading frames (ORF) revealed only one complete ORF of 1479 bp. There was no typical ribosome-binding site but only putative Epsilon and Omega enhancer sequences, similar to other reported carrageenases [7], [16]. A putative promoter sequence, TTAGCA for the −35 region and TATAAA for the −10 region, was present 136-bp upstream from the initiation codon with 20-bp spacing. An inverted repeat AAGCAGC and GCTGCTT with a TATTACTTTTGTAGT loop located 21-bp downstream of the TAG stop codon might function as a transcriptional termination site.

The deduced product, CgiA_Ce, of 492 amino acid residues had a theoretical molecular mass of 53.8 kDa and pI value of 9.5. The region M1 to S41 was estimated as putative signal peptide deduced by the N-terminal sequence. The theoretical molecular mass of the mature CgiA_Ce was 49.2 kDa which corresponds to the apparent molecular mass of the purified CgiA_Ce (48 kDa). Database search using the BLASTp program showed that this protein shared high sequence identity with the characterized ι-carrageenases from *Alteromonas fortis* and *Zobellia galactanovorans* (39% and 79%, respectively) [7]. Multiple alignment of family GH82 sequences (Figure 5A) showed that CgiA_Ce displayed the conserved catalytic residues of this family and the main secondary structures of CgiA_Af, including the flexible domain A [11]. All these data demonstrated that CgiA_Ce is indeed a new active member of family GH82.

### Consensus Sequence Analysis of the Active Site Residues of Family GH82

**Figure 6:**
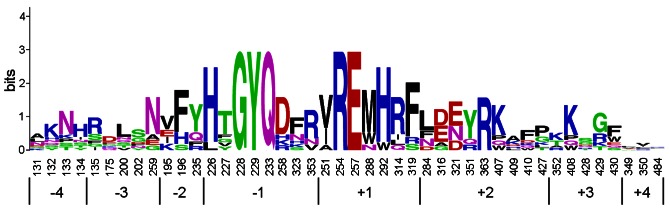
Sequence logo of the active site residues in family GH82. The abscissa numbers indicate the position of residues in CgiA_Ce. The logo was produced using the Weblogo server (http://weblogo.berkeley.edu/logo.cgi).

The sequence logo of the active site residues in family GH82 ι-carrageenases indicated that the residues at subsites −1 and +1 were more conserved than other subsite residues (Figure 6). Among the eight residues at subsite −1 and the seven residues at subsite +1, three highly conserved residues (Q233, E257 and H292 in CgiA_Ce) had been previously reported, explaining their crucial roles in catalysis [11], [12]. There were three additional strictly conserved residues, G228, Y229 and R254 in CgiA_Ce, at subsites −1 and +1, of which structural and functional roles have never been described before (Figure 5B). In this study, these three residues were chosen for site-directed mutagenesis to investigate their roles in family GH82 ι-carrageenases.

### Enzyme Specific Activity and Kinetic Parameters of rCgiA_Ce and its Mutants

**Figure 7:**
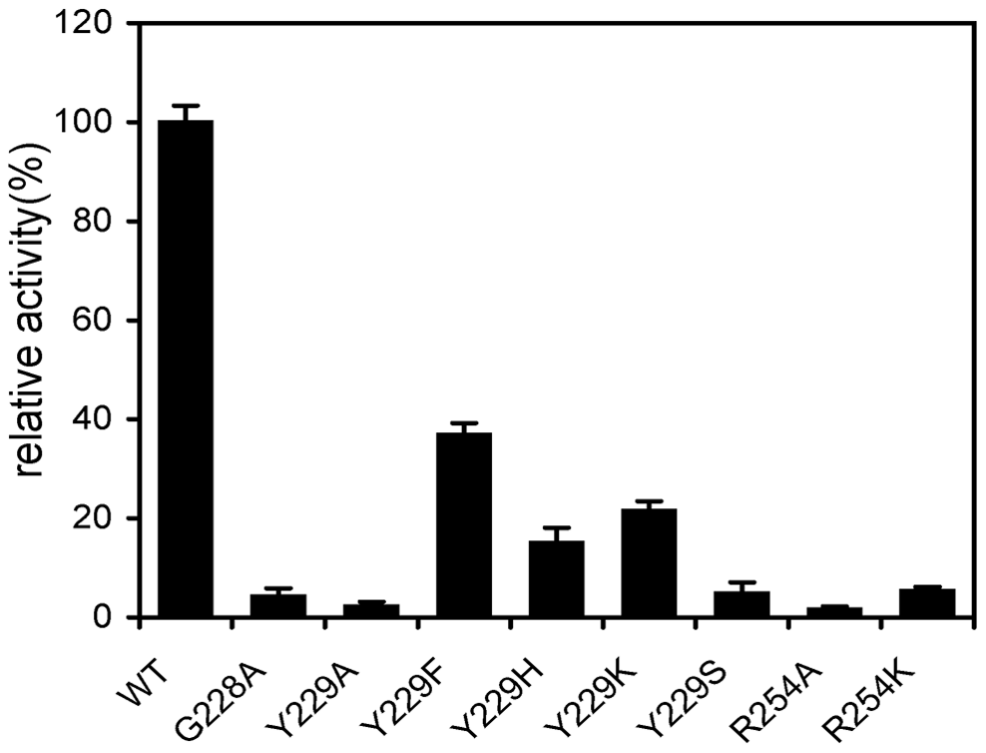
Relative activities of rCgiA_Ce and its mutants. WT: wild type.

To determine the importance of the three selected residues (G228, Y229 and R254), we initially mutated them into alanine. Moreover, each of the three residues was mutated to the structurally related amino acids to investigate their functional roles. The relative specific activities and kinetic parameters of rCgiA_Ce and its mutants were summarized in Figure 7 and Table 3.

**Table 3:** Kinetic Parameters of rCgiA_Ce and its mutants.

	Km (mM)	*kcat* (s^−1^)	*kcat*/Km (mM^−1^ s^−1^)
Wild type	3.68±0.094	14.22±0.23	3.86±0.036
Y229F	9.26±0.056	5.42±0.10	0.59±0.010
Y229K	6.72±0.076	2.93±0.18	0.44±0.023

When G228 was mutated to Ala, there was a 96% diminution of specific activity. Glycine has the simplest amino acid structure. The minimal steric hindrance of the glycine without a side chain allows much more structural flexibility than any other amino acids. The methyl side chain of the alanine residue might reduce the flexibility for enzyme active site and affect the enzyme-substrate interaction.

**Figure 8:**
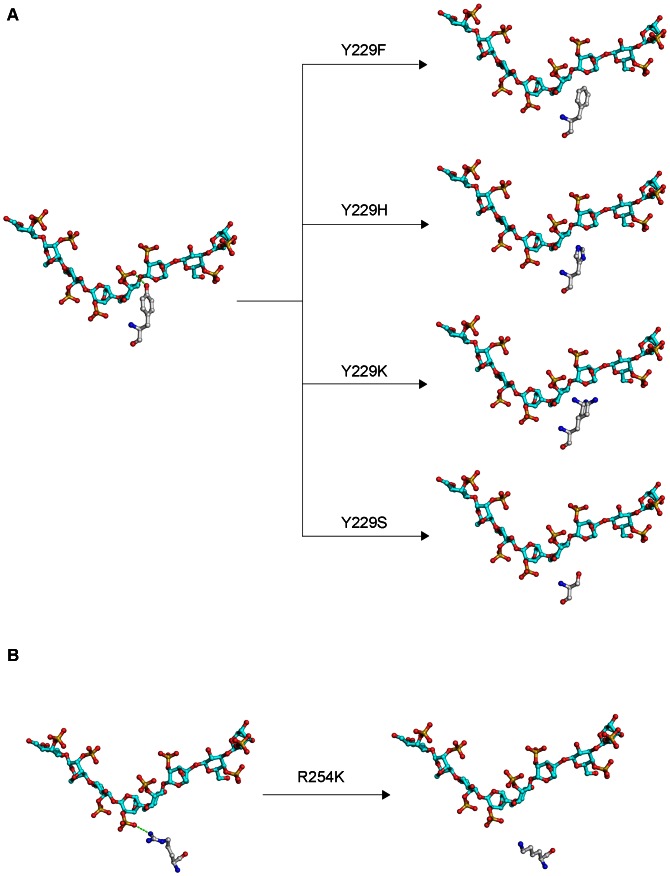
Structure modeling of specific residues of CgiA_Ce and their mutants complexed with carrageenan oligosaccharides. (A) Modeled structures of the Y229 and its mutants. (B) Modeled structures of the R254 and its mutant.

The Y229A mutant lost almost all ι-carrageenase activity, indicating that the Y229 in CgiA_Ce was one of the essential residues. For further investigation of its role, Y229 was replaced by phenyl- and hydroxyl-containing residues Phe and Ser (Figure 8A), respectively. The mutations resulted in significant losses of ι-carrageenase activity indicating that both the hydroxyl group and the phenyl ring were the key elements. Structure modeling of CgiA_Ce suggested that the hydroxyl group of Y229 extended towards the active site tunnel. Y229 was considered to contribute to the binding of neo-ι-carra-oligosaccharide by hydrogen bonding, as the distance between the hydroxyl of Y229 and the sulfate group of the G4S at subsite −1 was 2.8 Å (Figure 8A). Y229 was further replaced by His and Lys (Figure 8A) as these two residues contain positively charged amino groups which spatially substitute the hydroxyl of tyrosine and might form electrostatic interactions with the sulfate group of G4S. However, none of these mutants were active indicating that Y229 was un-replaceable at the active site of CgiA_Ce. Furthermore, the Km values for the mutants Y229F and Y229K apparently increased (Table 3), demonstrating that the mutations reduced the enzyme activities by weakening the binding of substrate to enzyme. Altogether, these results indicated that Y229 played a critical role in substrate binding in CgiA_Ce.

Based on the structure modeling of CgiA_Ce, the guanidine moiety of R254 was close to the sulfate group of A2S at subsite +1 (2.84 Å), indicating that it could interact with the substrate by ionic interaction. Mutant R254A produced an inactive enzyme, indicating the critical role of this residue. Moreover, R254K mutant resulted in a 94% loss of specific activity, indicating that both the length of the side chain and the guanidine moiety of the arginine residue were important.

## Discussion

The carrageenan oligosaccharides have been reported to have antiviral, antitumor, antioxidant and immunoloregulation activities [17]–[20]. Therefore, hydrolysis of carrageenan recently became a hot topic. However, carrageenan is gelatinous and viscous at the low temperature. High temperature leads to the transition of polysaccharide conformation followed by dissociation of helices, allowing the enzyme more accessible to the substrate in order to act on the polysaccharide. Unfortunately, only a few thermostable carrageenases have been found, limiting the application of carrageenases in the production of oligosaccharides. It has been reported that the κ-carrageenase from *Tamlana* sp. HC4 was stable below 45°C [21] and the κ-carrageenase CgkP from *Pseudoalteromonas* sp. QY203 remained 70% of original activity after incubation at 40°C for 48 h [22]. However, more than 60% of their activities were lost after incubation at 50°C for 1 h [21], [22]. There has no report on the thermostabilities of ι- and λ-carrageenases. We have performed here a comprehensive study on the biochemical characterization of the ι-carrageenase CgiA_Ce. To the best of our knowledge, CgiA_Ce is the most thermostable carrageenases reported to date. The prominent thermal stability will certainly find a wider industrial application of ι-carrageenase CgiA_Ce.

In addition to the catalytic residues, glycoside hydrolases often contain multiple substrate-binding residues in the active site which are beneficial for enzyme efficiency because they could assist the enzyme adhering to the substrate. For a polyanionic polysaccharide, such as carrageenan, basic amino acid residues in the enzyme are important for binding to the substrate by electrostatic interactions [6], [11]. The sequence logo (Figure 6) shows that all the eight subsites of family GH82 active site have basic amino acid residues (in blue color). The basic arginine residues at position 254 and 314 in CgiA_Ce are close (∼3 Å) to the sulfated monosaccharide A2S at subsite +1, indicating that they can both interact with the substrate by electrostatic interactions, consistent with the crystal structure of CgiA_Af [11]. The enzyme activities after R254A and R314A mutations were almost completely abolished (Figure S3), corroborating that they are both essential for CgiA_Ce. Furthermore, in family GH82, the arginine residue at position 254 is more conserved than that at position 314. The mutant R254K affected the enzyme activity more than R314K indicating that the conserved arginine is more essential for the enzyme (Figure S3).

We report here, for the first time, that Y229 in CgiA_Ce is a fully conserved and pivotal substrate-binding residue in family GH82. Among all the residues, Phe, Ser, Lys and His have some structural similarities to Tyr. Removal of either the hydroxyl group (Y229F) or the phenyl ring (Y229S), or mutating to a basic amino acid (Y229H or Y229K), significantly reduced the enzyme activities. These results shed light on the importance of this conserved residue in family GH82. Based on the CgiA_Ce structural model, the hydroxyl group of Y229 can interact with the sulfate group of G4S at subsite −1 by hydrogen bonding, but the aromatic ring does not directly interact with the substrate. The aromatic ring is stabilized by G228 and the side chain of I194 through hydrophobic interactions and maintains the right orientation and distance for the hydroxyl group of Y229 to interact with G4S. Moreover, the multiple sequence alignment showed that Y229 was flanked by glycine residues which were strictly conserved in family GH82 (Figure S4). Glycine provides a great flexibility necessary for the enzyme active sites to adopt the right conformation [23]. Thus, we tend to consider that the two flanking glycine residues at subsite −1 in family GH82 provide the space and flexibility for the tyrosine to interact with the substrate.

Enzymes are usually very specific to their substrates. Substrates bind to enzymes through hydrogen bonds, hydrophobic interactions, temporary covalent interactions or a combination of all of these, to form the enzyme-substrate complexes. Residues of the active sites, especially the substrate-binding residues, play important roles in recognizing the specific substrates. It has been reported that two key residues W443 and Y482 at subsite +1 of GH20-1 may determine the β-1,2 substrate specificity of family GH20 β-N-acetylhexosaminidase [24]. For endo-β-1,4-glucanase (GH12), a stacking interaction of hydrophobic nature between Y24 and the xylose side chain is crucial for xyloglucan recognition instead of carboxylmethylated 1,4-glucan [25]. κ-Carrageenan has the same backbone as ι-carrageenan but lacks the sulfate group in the A-unit (Figure S5). However, family GH82 ι-carrageenases have no activity towards κ-carrageenan. Based on our results, Y229 and R254 in CgiA_Ce interact with the sulfate groups of G4S (−1) and A2S (+1) of ι-carrageenan, respectively. The arginine at subsite +1 cannot interact with the α-Gal of κ-carrageenan likely weakening enzyme-substrate binding. In addition, there are no other strong interaction between the amino acid residues and the sugar ring at subsite +1 of family GH82. Taken together, we consider that the conserved substrate-binding residues at subsites −1 and +1 may determine the substrate specificity of family GH82 toward ι-carrageenan.

## Materials and Methods

### Bacterial Strains, Plasmids and Culture Conditions

*Cellulophaga* sp. QY3 was isolated from the red algae *Grateloupia livida* collected from Qingdao coastal water of China Yellow Sea and preserved in our laboratory. This area is a public place needed no specific permissions and the species in our studies aren't the endangered or protected species. This marine bacterium was isolated and grown at 25°C in a medium consisting of: 3% NaCl, 0.3% MgSO_4_•7H_2_O, 0.15% Na_2_HPO_4_, 0.1% NaH_2_PO_4_, 0.02% CaCl_2_, 0.01% KCl, 0.002% FeSO_4_, 0.3% casein, and 0.2% ι-carrageenan (w/v). *Escherichia coli* strains DH5α (Gibco BRL) and BL21 (DE3) (Novagen) containing pET-28a(+) were grown at 37°C in Luria-Bertani (LB) broth or on LB agar supplemented with kanamycin (30 µg/ml) when relevant.

### ι-Carrageenase Activity Assay

Aliquots (100 µl) of ι-carrageenase (0.01–0.1 mg/ml) were incubated with 900 µl of substrate solutions consisting of 0.2% (w/v) ι-carrageenan (type V, from *Eucheuma spinosa*, Sigma-Aldrich) in 20 mM phosphate buffer (pH 7.0) at 50°C for 20 min. The reducing oligosaccharide products in the reaction mixture were assayed using 3,5-dinitrosalicylic acid method [26]. One unit of ι-carrageenase activity was defined as the amount of enzyme required to liberate 1 µmol of reducing sugar as neo-ι-carrabiose per min under the above conditions.

### Purification of CgiA_Ce

Strain QY3 was cultured at 25°C for 20h. The purification of CgiA_Ce was carried out at 4°C. The culture medium was centrifuged (10,000 g for 10 min) and the cell-free culture supernatant was brought to 30% (w/v) with solid ammonium sulfate and stirred for 1 h. After centrifugation (13,000 g for 30 min), the supernatant was applied to a column of high-performance Phenyl Sepharose (1 cm×20 cm; GE Healthcare, UK) previously equilibrated with 50 mM phosphate buffer (pH 7.0) containing 30% (w/v) (NH_4_)_2_SO_4_. The column was washed with equilibration buffer to remove the unbound material and eluted with a linear gradient of (NH_4_)_2_SO_4_ from 30% to 0% over 200 ml at a flow rate of 1 ml/min. The active fractions were pooled and desalted using an Amicon concentrator with an ultrafiltration membrane (MWCO 10,000; Merck Millipore, Germany). The concentrate (4 ml) was loaded onto a preparative Superdex 75 HR 10/30 column (GE Healthcare, UK) equilibrated with 20 mM phosphate buffer (pH 7.0) containing 0.2 M NaCl, then eluted with the same buffer at a flow rate of 0.5 ml/min. The active fractions were pooled and desalted as described above. SDS-PAGE was performed as described by Laemmli [27]. Protein concentration was determined using a protein assay kit (Bio-Rad, USA) with BSA (Sigma-Aldrich, USA) as the standard.

### Biochemical Characterization of CgiA_Ce

The optimum temperature of the CgiA_Ce activity was determined by carrying out the enzyme activity assays at pH 7.0 and temperatures ranging from 0 to 70°C. The thermostability of the CgiA_Ce was evaluated by measuring the residual activities of the enzyme after incubation at pH 7.0 and different temperatures for 1 h. The pH effect on ι-carrageenase activity was determined under the standard assay conditions at 50°C in various buffers (50 mM): citrate buffer (pH 3.0–6.0), phosphate buffer (pH 6.0–8.0), Tris-HCl buffer (pH 7.5–9.0) and glycine-NaOH buffer (pH 8.6–10.6). To determine the pH stability of the CgiA_Ce, the residual enzyme activities were measured after incubation at 4°C in various buffers (pH 4.0–10.6) for 24 h. The effects of various metal ions and chelators on CgiA_Ce activities were examined by monitoring enzymatic activities in the presence of 1 mM of various cation ions or chelators. The substrate specificity of the CgiA_Ce was determined using ι-carrageenan, κ-carrageenan, λ-carrageenan, agar, alginate, cellulose and starch (Sigma-Aldrich, USA) as the substrates, respectively.

### Analysis of Hydrolytic Pattern and Products of CgiA_Ce

The hydrolysis reaction of ι-carrageenan (0.2%, w/v; 100 ml) with CgiA_Ce (5 U) at 45°C was monitored for up to 72 h. An aliquot (0.1 ml) of hydrolysis product was taken out at different times and analyzed using fluorophore-assisted carbohydrate electrophoresis (FACE) [28]. To visualize the polysaccharide and the oligosaccharide products, the gels were stained in a 0.2% (w/v) Alcian Blue solution for 3 h and destained with 2% (w/v) acetic acid.

To identify the structure of hydrolysis products, oligosaccharides with different DP were fractionated on a Bio-Gel P6 column (16 mm×96 cm, 25°C; Bio-Rad, USA) [28]. NH_4_HCO_3_ (0.2 M) was used as eluent at a flow rate of 6 ml/h. The eluted fractions were detected using the phenol-H_2_SO_4_ method. The NH_4_HCO_3_ salt was removed by repeated co-evaporation with water under diminished pressure at 50°C. ^1^H- and ^13^C-NMR were carried out on a JNMECP600 (JEOL) instrument, with 2,2-dimethyl-2-silapentane-5-sulfonic acid as the internal standard. Molecular masses of the oligosaccharide products were determined by negative-ion ESI-MS performed on a LTQ Orbitrap XL mass spectrometer (ThermoFisher Scientific, USA). I spray voltage: 3 kV; tube lens: −80 V; capilary temp: 275°C; capilary voltage: −43 V; sheath gas flow rate: 8 arb. Samples were dissolved in CH_3_CN/H_2_O (1∶1, v/v), typically at a concentration of 5–10 pmol/µl, of which 5 µl was loop-injected. The data were collected and processed by Xcalibur software.

### Substrate Binding Subsites of CgiA_Ce

To determine the smallest substrate and the number of substrate binding subsites in its catalytic tunnel of CgiA_Ce, hydrolysis reactions were carried out using oligosaccharides with different chain lengths at a concentration of 10 mg/ml in 10 µl of reaction mixture (pH 7.0). According to the different DPs of oligosaccharides, the substrate solutions were mixed with different amounts of CgiA_Ce and incubated at 45°C for different times. Hydrolysis products were analyzed by FACE.

To determine the direction of the substrate in the active site of CgiA_Ce, the reducing end of the hexasaccharide was fluorescently labeled using AGA reagents by reductive-amination [28]. The AGA-labeled hexasaccharide was purified on a Sephadex G10 column (10 mm×50 cm; GE Healthcare, UK), then degraded by CgiA_Ce. The degradation product was subjected to PAGE (22%, w/v) analysis [7].

### Sequencing of the N-Terminal and Internal Regions of CgiA_Ce

The N-terminal sequence of the purified CgiA_Ce was determined by automated Edman degradation with a protein sequencer (ProciseTM 491, Applied Biosystems, USA). In addition, LC-ESI-MS/MS analysis of the tryptic peptides was carried out on a LTQ Orbitrap instrument for *de novo* sequencing.

### Cloning of the ι-Carrageenase Gene

Genomic DNA of *Cellulophaga* sp. QY3 was isolated using the Genomic DNA Extraction Kit (Takara, Dalian, China) according to the manufacturer’s instructions and used as the template of degenerate PCR. Degenerate primers (PcgiA-F1 and PcgiA-R1; Table S1) were designed corresponding to the peptide sequences obtained above. A 0.9 kb DNA fragment was obtained and sequenced. To obtain the remainder of the *cgiA_Ce* gene, an inverse PCR was carried out using primers (PcgiA-F2,3 and PcgiA-R2,3; Table S1) designed according to the obtained 921-bp degenerate PCR product. The inverse PCR products were purified, sequenced, and assembled with the initial *cgiA_Ce* fragment to obtain the full-length gene.

### Bioinformatics Analysis

The putative translation frame was identified by the DNATools program, and the theoretical molecular mass and isoelectric point were calculated using the Compute pI/Mw tool (http://web.expasy.org/compute_pi). A functional domain search was performed to determine the protein family and domain organization using the Pfam search server (http://www.sanger.ac.uk/Software/Pfam) and the NCBI BLAST server (http://blast.ncbi.nlm.nih.gov/Blast.cgi). Pairwise and multiple sequence alignments between CgiA_Ce and other known ι-carrageenases were obtained using the CLUSTAL X program [29]. SWISS-MODEL software (http://swissmodel.expasy.org/) was performed to create the 3D homology models of CgiA_Ce and its derivatives using the crystal structure of CgiA_Af (PDB accession code 1KTW) as the template [30]. The PyMol molecular Graphics System (DeLano Scientific, San Carlos, USA. http://www.pymol.org) was used to visualize and analyze the modeled structure and to construct graphical presentations and illustrative figures. Using the crystal structure of CgiA_Af complexed with neo-ι-carratetraose and neo-ι-carrabiose (PDB accession code 1KTW) as a guide, a neo-ι-carrabiose with the same configuration was manually docked into the subsites −1 and −2 of modeled structure of CgiA_Ce and positioned.

### Consensus Sequence Analysis of the Active Site Residues of Family GH82

The amino acid residues around the active site, especially within 6 Å around the substrate according to the crystal structure of the CgiA_Af, were selected. The selected amino acid residues of all members of family GH82 ι-carrageenases were obtained by multiple sequence alignment and further used for generation of sequence logo by WebLogo (Version 2.8.2) [31]. A sequence logo shows the relative frequencies of the various residues at a given position. This is indicated by proportionally varying the size of the symbol. The order of predominance of the residues at a given position is indicated by showing the most frequently occurring residue at the top of the heap and least frequently occurring residue at the bottom of the heap. The height of the logo at a given position is proportional to the degree of conservation at that position.

### Expression and Purification of Wild Type rCgiA_Ce and its Mutants

The *cgiA_Ce* gene, including its signal sequence, was PCR-amplified from genomic DNA using primers PcgiA-F4 and PcgiA-R4 (Table S1) to introduce the *Bam*H I and *Xho* I sites, respectively. The PCR product was purified, digested with *Bam*H I and *Xho* I, and ligated into the similarly digested pET-28a(+) expression vector to generate plasmid pET28-cgiA. Site-directed mutagenesis was carried out using the TaKaRa MutanBEST Kit (TaKaRa, Dalian, China) according to the manufacturer’s instruction. Primers used in the site-directed mutagenesis study were presented in Table S2. All constructs were transformed into *E. coli* BL21 (DE3) for protein expression.

The *E. coli* BL21 (DE3) cells harboring either pET28-cgiA or its mutants were grown at 37°C in LB medium containing 30 µg/ml kanamycin until the optical density at 600 nm reached 1.0 before addition of isopropylthio-β-galactopyranoside (IPTG) to a final concentration of 0.7 mM. The cultivation was continued further for 36 h at 16°C and 100 rpm. *E. coli* BL21 (DE3) bearing pET-28a(+) was used as the negative control. The culture supernatant, obtained by centrifugation (10,000 g for 10 min), was dialysed against phosphate buffer (20 mM, pH 7.5; 500 mM NaCl) and loaded onto a HiTrap HP column (1 ml; GE Healthcare, UK). The recombinant proteins were eluted by a linear gradient of imidazole (5–500 mM) in phosphate buffer (20 mM, pH 7.5; 500 mM NaCl). Active fractions were estimated according to SDS-PAGE analysis as described above.

### Kinetic Parameters of Wild Type rCgiA_Ce and its Mutants

The initial velocities (v_0_) were determined by measuring the activities at 3–6 different time points. Substrate concentrations over a range of approximately 0.2 to 5 times of the apparent Km were used to determine the kinetic parameters. Purified enzyme (100 µl) was incubated with 900 µl substrates of different concentrations in phosphate buffer (pH 7.0) at 50°C for 3 min. All measurements were performed in triplicate. The apparent kinetic parameters including *kcat*, Km, *kcat*/Km were obtained from Lineweaver–Burk plots, which were assessed by using a standard linear regression function.

### Nucleotide Sequence Accession Numbers

The nucleotide sequence of *cgiA_Ce* and its flanking region has been submitted to the GenBank database under accession number of JX871894.

## Supporting Information

Figure S1 **Gel-filtration on Bio-gel P6 of the neo-ι-carra-oligosaccharides derived from CgiA_Ce degradation.**
(DOC)

Figure S2 **NMR spectra of the neo-ι-carratetraose.** (A) ^1^H NMR spectrum of neo-ι-carratetraose. (B) ^13^C NMR spectrum of neo-ι-carratetraose.
(DOC)

Figure S3 **Relative activities of rCgiA_Ce and mutants of R254 and R314.** WT: wild type
(DOC)

Figure S4 **Multiple sequence alignment of the family GH82 carrageenases.**
(DOC)

Figure S5 **Schematic representation of the different structures of the repeating dimeric units of κ- and ι-carrageenan.**
(DOC)

Table S1 **List of primers used in this study.**
(DOC)

Table S2 **Oligonucleotides used to mutagenize the CgiA_Ce.**
(DOC)

## References

[1] Popper ZA, Michel G, Hervé C, Domozych DS, Willats WG, et al (2011) Evolution and diversity of plant cell walls: from algae to flowering plants. Ann Rev Plant Biol 62: 567–590. 21351878 10.1146/annurev-arplant-042110-103809

[2] Yang B, Yu G, Zhao X, Jiao G, Ren S, et al (2009) Mechanism of mild acid hydrolysis of galactan polysaccharides with highly ordered disaccharide repeats leading to a complete series of exclusively odd-numbered oligosaccharides. FEBS J 276: 2125–2137. 19292880 10.1111/j.1742-4658.2009.06947.x

[3] Kilor VA, Sapkal NP, Awari JG, Shewale BD (2010) Development and characterization of enteric-coated immediate-release pellets of aceclofenac by extrusion/spheronization technique using kappa-carrageenan as a pelletizing agent. AAPS PharmSciTech 11: 336–343. 20195805 10.1208/s12249-010-9389-9 PMC2850460

[4] Siepmann F, Muschert S, Zach S, Leclercq B, Carlin B, et al (2007) Carrageenan as an efficient drug release modifier for ethylcellulose-coated pharmaceutical dosage forms. Biomacromolecules 8: 3984–3991. 18039003 10.1021/bm7009587

[5] Michel G, Nyval-Collen P, Barbeyron T, Czjzek M, Helbert W (2006) Bioconversion of red seaweed galactans: a focus on bacterial agarases and carrageenases. Appl Microbiol Biotechnol 71: 23–33. 16550377 10.1007/s00253-006-0377-7

[6] Michel G, Chantalat L, Duee E, Barbeyron T, Henrissat B, et al (2001) The kappa-carrageenase of *P. carrageenovora* features a tunnel-shaped active site: a novel insight in the evolution of Clan-B glycoside hydrolases. Structure 9: 513–525. 11435116 10.1016/s0969-2126(01)00612-8

[7] Barbeyron T, Michel G, Potin P, Henrissat B, Kloareg B (2000) iota-Carrageenases constitute a novel family of glycoside hydrolases, unrelated to that of kappa-carrageenases. J Biol Chem 275: 35499–35505. 10934194 10.1074/jbc.M003404200

[8] Guibet M, Colin S, Barbeyron T, Genicot S, Kloareg B, et al (2007) Degradation of lambda-carrageenan by *Pseudoalteromonas carrageenovora* lambda-carrageenase: a new family of glycoside hydrolases unrelated to kappa- and iota-carrageenases. Biochem J 404: 105–114. 17269933 10.1042/bj20061359 PMC1868830

[9] Cantarel BL, Coutinho PM, Rancurel C, Bernard T, Lombard V, et al (2009) The Carbohydrate-Active EnZymes database (CAZy): an expert resource for Glycogenomics. Nucleic Acids Res 37: D233–238. 18838391 10.1093/nar/gkn663 PMC2686590

[10] Hatada Y, Mizuno M, Li Z, Ohta Y (2010) Hyper-production and characterization of the ι-carrageenase useful for ι-carrageenan oligosaccharide production from a deep-sea bacterium, *Microbulbifer thermotolerans* JAMB-A94T, and insight into the unusual catalytic mechanism. Mar Biotechnol (NY) 13: 411–422. 20686828 10.1007/s10126-010-9312-0

[11] Michel G, Helbert W, Kahn R, Dideberg O, Kloareg B (2003) The structural bases of the processive degradation of iota-carrageenan, a main cell wall polysaccharide of red algae. J Mol Biol 334: 421–433. 14623184 10.1016/j.jmb.2003.09.056

[12] Rebuffet E, Barbeyron T, Jeudy A, Jam M, Czjzek M, et al (2010) Identification of catalytic residues and mechanistic analysis of family GH82 iota-carrageenases. Biochemistry 49: 7590–7599. 20681629 10.1021/bi1003475

[13] Lemoine M, Nyvall Collén P, Helbert W (2009) Physical state of kappa-carrageenan modulates the mode of action of kappa-carrageenase from *Pseudoalteromonas carrageenovora* . Biochem J 419: 545–553. 19196238 10.1042/BJ20080619

[14] Jouanneau D, Boulenguer P, Mazoyer J, Helbert W (2010) Complete assignment of ^1^H and ^13^C NMR spectra of standard neo-iota-carrabiose oligosaccharides. Carbohydr Res 345: 547–551. 20038459 10.1016/j.carres.2009.12.004

[15] Ma C, Lu X, Shi C, Li J, Gu Y, et al (2007) Molecular cloning and characterization of a novel beta-agarase, AgaB, from marine *Pseudoalteromonas* sp. CY24. J Biol Chem 282: 3747–3754. 17166842 10.1074/jbc.M607888200

[16] Barbeyron T, Gerard A, Potin P, Henrissat B, Kloareg B (1998) The kappa-carrageenase of the marine bacterium *Cytophaga drobachiensis*. Structural and phylogenetic relationships within family-16 glycoside hydrolases. Mol Biol Evol 15: 528–537. 9580981 10.1093/oxfordjournals.molbev.a025952

[17] Kalitnik AA, Byankina Barabanova AO, Nagorskaya VP, Reunov AV, Glazunov VP, et al (2013) Low molecular weight derivatives of different carrageenan types and their antiviral activity. J Appl Phycol 25: 65–72.

[18] Wang W, Zhang P, Hao C, Zhang XE, Cui ZQ, et al (2011) In vitro inhibitory effect of carrageenan oligosaccharide on influenza A H1N1 virus. Antiviral Res 92: 237–246. 21867732 10.1016/j.antiviral.2011.08.010

[19] Chen H, Yan X, Lin J, Wang F, Xu W (2007) Depolymerized products of lambda-carrageenan as a potent angiogenesis inhibitor. J Agric Food Chem 55: 6910–6917. 17661479 10.1021/jf070183+

[20] Xu L, Yao Z, Wu H, Wang F, Zhang S (2012) The immune regulation of κ-carrageenan oligosaccharide and its desulfated derivatives on LPS-activated microglial cells. Neurochem Int 61: 689–696. 22766495 10.1016/j.neuint.2012.06.019

[21] Sun F, Ma Y, Wang Y, Liu Q (2010) Purification and characterization of novel κ-carrageenase from marine *Tamlana* sp. HC4. Chin J Oceanol Limnol 28: 1139–1145.

[22] Li S, Jia P, Wang L, Yu W, Han F (2013) Purification and characterization of a new thermostable κ-carrageenase from the marine bacterium *Pseudoalteromonas* sp. QY203. J Ocean Univ China 12: 155–159.

[23] Yan BX, Sun YQ (1997) Glycine residues provide flexibility for enzyme active sites. J Biol Chem 272: 3190–3194. 9013553 10.1074/jbc.272.6.3190

[24] Jiang YL, Yu WL, Zhang JW, Frolet C, Di Guilmi AM, et al (2011) Structural basis for the substrate specificity of a novel beta-N-acetylhexosaminidase StrH protein from *Streptococcus pneumoniae* R6. J Biol Chem 286: 43004–43012. 22013074 10.1074/jbc.M111.256578 PMC3234876

[25] Yoshizawa T, Shimizu T, Hirano H, Sato M, Hashimoto H (2012) Structural basis for inhibition of xyloglucan-specific endo-beta-1,4-glucanase (XEG) by XEG-protein inhibitor. J Biol Chem 287: 18710–18716. 22496365 10.1074/jbc.M112.350520 PMC3365752

[26] Miller GL (1959) Use of dinitrosalicylic acid reagent for determination of reducing sugar. Anal Chem 31: 426–428.

[27] Laemmli UK (1970) Cleavage of structural proteins during the assembly of the head of bacteriophage T4. Nature 227: 680–685. 5432063 10.1038/227680a0

[28] Li J, Han F, Lu X, Fu X, Ma C, et al (2007) A simple method of preparing diverse neoagaro-oligosaccharides with beta-agarase. Carbohydr Res 342: 1030–1033. 17359946 10.1016/j.carres.2007.02.008

[29] Thompson JD, Gibson TJ, Plewniak F, Jeanmougin F, Higgins DG (1997) The CLUSTAL_X windows interface: flexible strategies for multiple sequence alignment aided by quality analysis tools. Nucleic Acids Res 25: 4876–4882. 9396791 10.1093/nar/25.24.4876 PMC147148

[30] Schwede T, Kopp J, Guex N, Peitsch MC (2003) SWISS-MODEL: An automated protein homology-modeling server. Nucleic Acids Res 31: 3381–3385. 12824332 10.1093/nar/gkg520 PMC168927

[31] Crooks GE, Hon G, Chandonia JM, Brenner SE (2004) WebLogo: a sequence logo generator. Genome Res 14: 1188–1190. 15173120 10.1101/gr.849004 PMC419797

