# Supplementary material for: Purification, Cloning, Characterization and Essential Amino Acid Residues Analysis of a New ι-Carrageenase from Cellulophaga sp. QY3
Source: PLoS One. 2013 May 31;8(5):e64666. doi: 10.1371/journal.pone.0064666 (PMC3669377; doi:10.1371/journal.pone.0064666)
Supplement: Figure S1 — Gel-filtration on Bio-gel P6 of the neo-ι-carra-oligosaccharides derived from CgiA_Ce degradation. (DOC) [file pone.0064666.s001.doc]

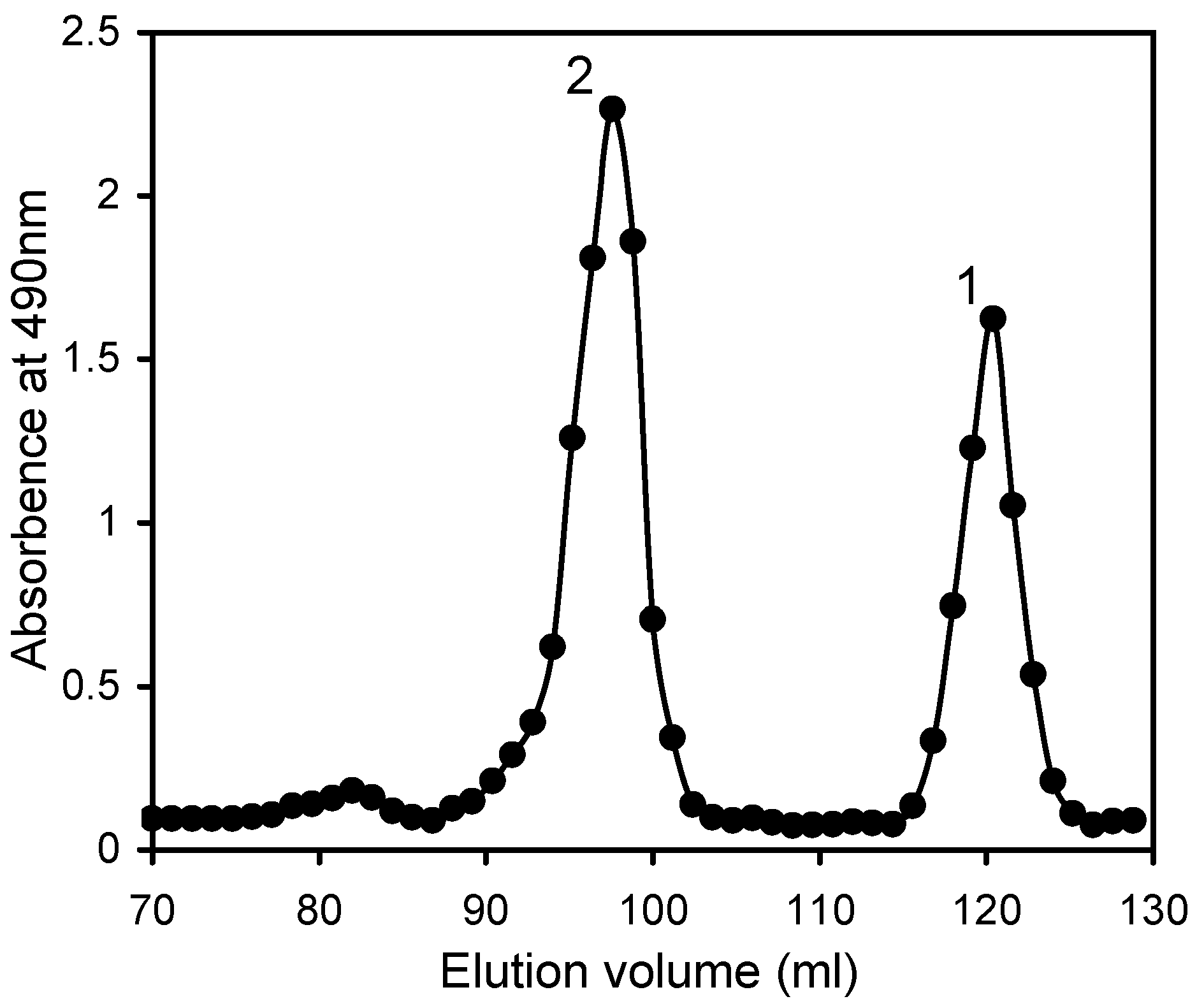


**Figure S1.** Gel-ﬁltration on Bio-gel P6 of the neo-ι-carra-oligosaccharides derived from CgiA_Ce degradation.
