# Supplementary material for: Purification, Cloning, Characterization and Essential Amino Acid Residues Analysis of a New ι-Carrageenase from Cellulophaga sp. QY3
Source: PLoS One. 2013 May 31;8(5):e64666. doi: 10.1371/journal.pone.0064666 (PMC3669377; doi:10.1371/journal.pone.0064666)
Supplement: Figure S2 — NMR spectra of the neo-ι-carratetraose. (A) 1H NMR spectrum of neo-ι-carratetraose. (B) 13C NMR spectrum of neo-ι-carratetraose. (DOC) [file pone.0064666.s002.doc]

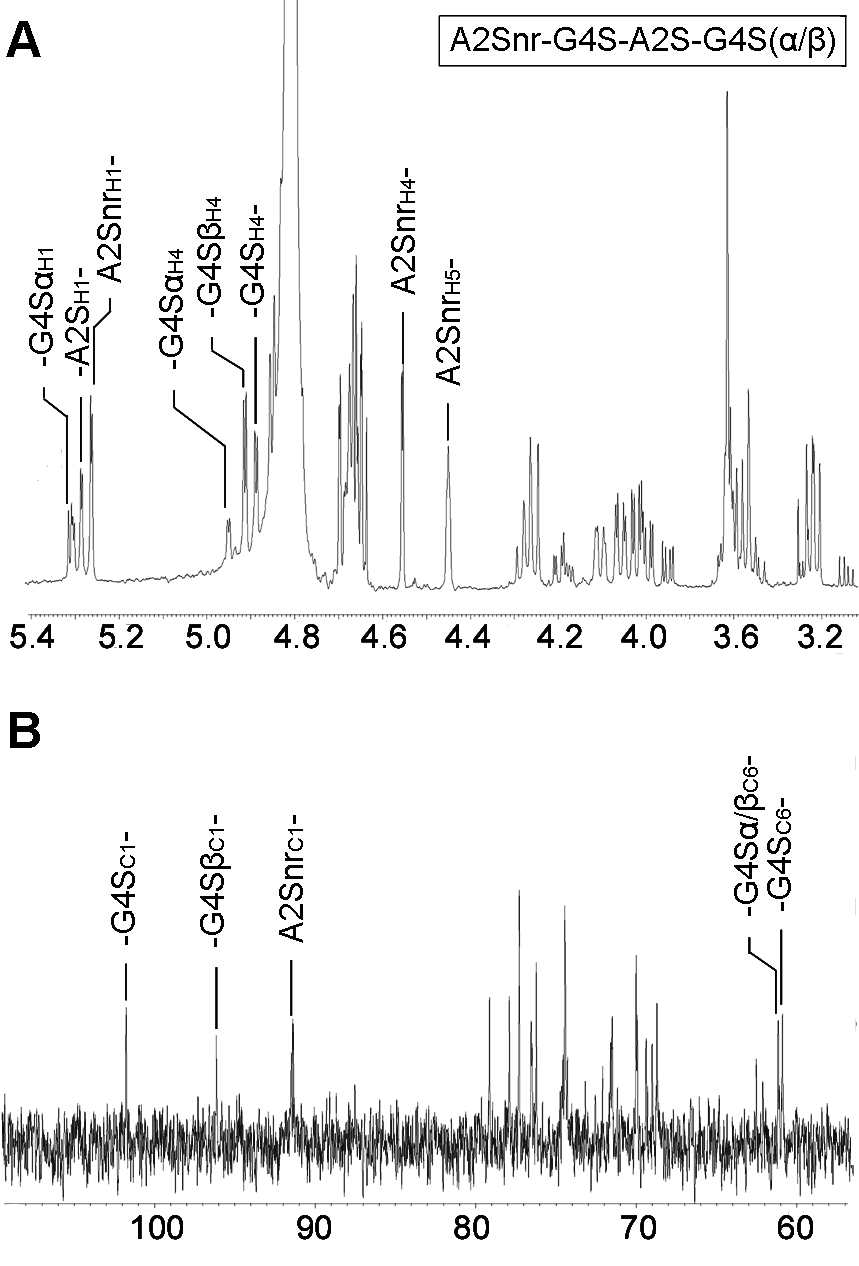


**Figure S2.** NMR spectra of the neo-ι-carratetraose. (A) ^1^H NMR spectrum of neo-ι-carratetraose. (B) ^13^C NMR spectrum of neo-ι-carratetraose.
