# Supplementary figures and images for: Purification, Cloning, Characterization and Essential Amino Acid Residues Analysis of a New ι-Carrageenase from Cellulophaga sp. QY3
Source: PLoS One. 2013 May 31;8(5):e64666. doi: 10.1371/journal.pone.0064666 (PMC3669377; doi:10.1371/journal.pone.0064666)

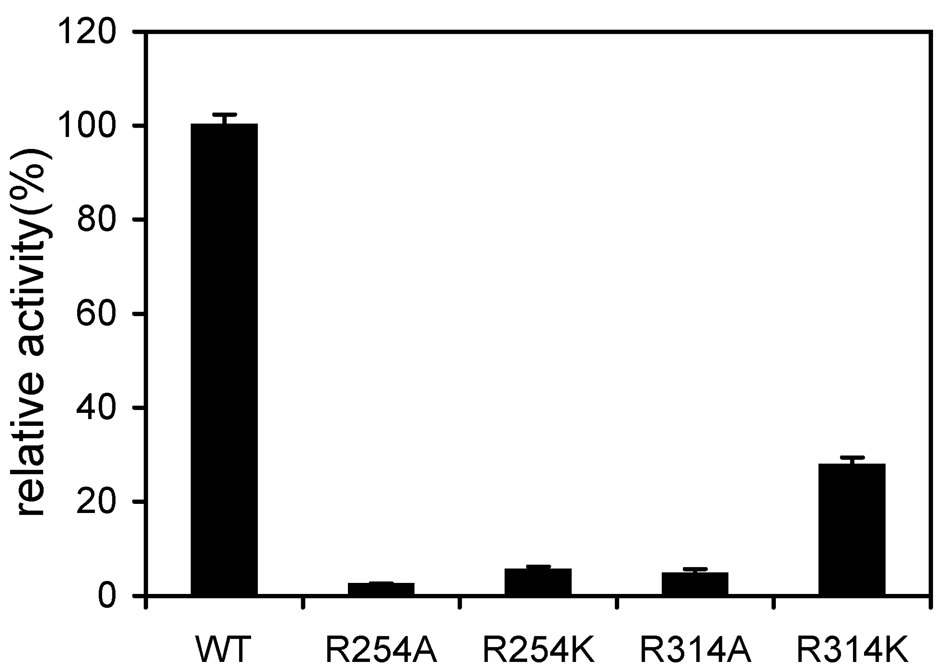


**Figure S3.** Relative activities of rCgiA_Ce and mutants of R254 and R314. WT: wild type

Supplement: Figure S3 — Relative activities of rCgiA_Ce and mutants of R254 and R314. WT: wild type (DOC) [file pone.0064666.s003.doc]

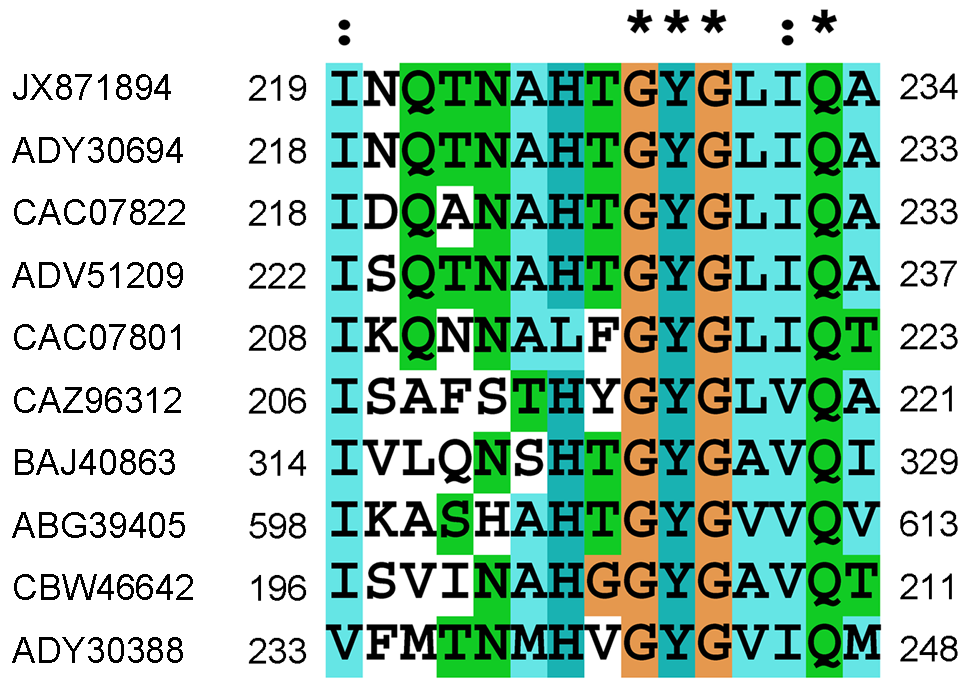


**Figure S4.** Multiple sequence alignment of the family GH82 carrageenases.

Supplement: Figure S4 — Multiple sequence alignment of the family GH82 carrageenases. (DOC) [file pone.0064666.s004.doc]
