# Supplementary material for: Purification, Cloning, Characterization and Essential Amino Acid Residues Analysis of a New ι-Carrageenase from Cellulophaga sp. QY3
Source: PLoS One. 2013 May 31;8(5):e64666. doi: 10.1371/journal.pone.0064666 (PMC3669377; doi:10.1371/journal.pone.0064666)
Supplement: Table S1 — List of primers used in this study. (DOC) [file pone.0064666.s006.doc]

**Table S1.** List of primers used in this study.

| Name of primers | Sequences of primers |
| --- | --- |
| PcgiA-F1 | 5’-AAYTTYTAYAARCCNCC-3’ |
| PcgiA-R1 | 5’-YTTRTANACNACYTCNAC-3’ |
| PcgiA-F2 | 5’-GCATCACCTGCTTTATTTGTA-3’ |
| PcgiA-R2 | 5’-TGTTATGTTCTCCCCTCACTT-3’ |
| PcgiA-F3 | 5’-ACACCACCAGACCCAGAAAT-3’ |
| PcgiA-R3 | 5’-AGCAGACGTTGGTAATGTAG-3’ |
| PcgiA-F4 | 5’-GGATCCATGATGAAACTAAAAATTAAACATG-3’ |
| PcgiA-R4 | 5’-CTCGAGATTACATGAATTTATTGTACCAAC-3’ |
