# Supplementary material for: Purification, Cloning, Characterization and Essential Amino Acid Residues Analysis of a New ι-Carrageenase from Cellulophaga sp. QY3
Source: PLoS One. 2013 May 31;8(5):e64666. doi: 10.1371/journal.pone.0064666 (PMC3669377; doi:10.1371/journal.pone.0064666)
Supplement: Table S2 — Oligonucleotides used to mutagenize the CgiA_Ce. (DOC) [file pone.0064666.s007.doc]

**Table S2.** Oligonucleotides used to mutagenize the CgiA_Ce.

| Mutant | Name of primers | Primers 5'-3' |
| --- | --- | --- |
| G228A | Pmu-F1 | **GCT**TATGGTTTAATACAAGCATATGCT |
|  | Pmu-R1 | TGTATGCGCATTTGTTTGGTTTAT |
| Y229A | Pmu-F2 | **GCT**GGTTTAATACAAGCATATGCTG |
|  | Pmu-R2 | ACCTGTATGCGCATTTGTTTGG |
| Y229F | Pmu-F3 | **TTT**GGTTTAATACAAGCATATGCTG |
| Y229S | Pmu-F4 | **TCT**GGTTTAATACAAGCATATGCTG |
| Y229H | Pmu-F5 | **CAT**GGTTTAATACAAGCATATGCTG |
| Y229K | Pmu-F6 | **AAA**GGTTTAATACAAGCATATGCTGC |
| R254A | Pmu-F7 | **GCA**TTAGAAACTGATAACCTTGCAAT |
|  | Pmu-R3 | TAAGGTTACACCACCTGTACAA |
| R254K | Pmu-F8 | **AAA**TTAGAAACTGATAACCTTGCAAT |

In each case, the mutated nucleotides are highlighted in bold.
